# Chemical Authentication of Botanical Ingredients: A Review of Commercial Herbal Products

**DOI:** 10.3389/fphar.2021.666850

**Published:** 2021-04-15

**Authors:** Mihael Cristin Ichim, Anthony Booker

**Affiliations:** ^1^ “Stejarul” Research Centre for Biological Sciences, National Institute of Research and Development for Biological Sciences, Piatra Neamt, Romania; ^2^ Research Centre for Optimal Health, School of Life Sciences, College of Liberal Arts and Sciences, University of Westminster, London, United Kingdom; ^3^ Pharmacognosy and Phytotherapy, UCL School of Pharmacy, London, United Kingdom

**Keywords:** chemical marker, natural product, herbal product, food supplement, herbal medicine, authentication, adulteration, contamination

## Abstract

Chemical methods are the most important and widely used traditional plant identification techniques recommended by national and international pharmacopoeias. We have reviewed the successful use of different chemical methods for the botanical authentication of 2,386 commercial herbal products, sold in 37 countries spread over six continents. The majority of the analyzed products were reported to be authentic (73%) but more than a quarter proved to be adulterated (27%). At a national level, the number of products and the adulteration proportions varied very widely. Yet, the adulteration reported for the four countries, from which more than 100 commercial products were purchased and their botanical ingredients chemically authenticated, was 37% (United Kingdom), 31% (Italy), 27% (United States), and 21% (China). Simple or hyphenated chemical analytical techniques have identified the total absence of labeled botanical ingredients, substitution with closely related or unrelated species, the use of biological filler material, and the hidden presence of regulated, forbidden or allergenic species. Additionally, affecting the safety and efficacy of the commercial herbal products, other low quality aspects were reported: considerable variability of the labeled metabolic profile and/or phytochemical content, significant product-to-product variation of botanical ingredients or even between batches by the same manufacturer, and misleading quality and quantity label claims. Choosing an appropriate chemical technique can be the only possibility for assessing the botanical authenticity of samples which have lost their diagnostic microscopic characteristics or were processed so that DNA cannot be adequately recovered.

## Introduction

Herbal products are being sold under many and diverse commercial descriptions in the international marketplace, including herbal drugs, botanical drugs, botanicals, phytomedicines, traditional medicines (TMs), herbal medicines (HMs), traditional herbal medicines products (THMPs), natural health products (NHPs), dietary supplements (DSs), plant food supplements (PFSs), nutraceuticals (NCs) and food supplements (FSs) (Ichim, 2019), the differences being mainly due to the prevailing national legislation under which they are marketed (Simmler et al., 2018). Herbal products are commercialized as medicines or foods, according to their officially declared intended final use by their manufacturers operating under various regulatory frameworks, and they are purchased, and subsequently used and consumed, for their medicinal claims (herbal medicines) or their expected health benefits (food supplements) (Thakkar et al., 2020). In the United Kingdom, for example, plant products are regulated under two main criteria, the first being what is claimed, i.e. if a manufacturer claims a medicinal effect, the product will automatically fall under medicines legislation; the second consideration being the activity of the plant *in vivo*, if it has shown to have a strong medicinal or pharmacological action then it is deemed a medicine regardless of the claims, the most notable plant in this category being *Hypericum perforatum* L. (St John’s Wort). Whereas in the United States most plant products are regulated as food supplements (botanicals) and in Germany the majority are considered medicines. Unfortunately, these marketing differences, due to significant differences between the regulatory approaches across jurisdictions (Low et al., 2017), are further contributing to their poor regulation on the international market.

Accidental contamination or the deliberate use of filler or substitute species (Shanmughanandhan et al., 2016) leads inherently to non-authentic, adulterated products (Simmler et al., 2018). The adulteration of commercial herbal products is an internationally widespread problem, as it has been reported for many countries from all inhabited continents (Ichim, 2019; Ichim et al., 2020). Moreover, large percentages of adulterated products have been reviewed, irrespective of the formal category of herbal products, being affected food and dietary supplements and medicines altogether (Ichim and de Boer, 2021), including products used in centuries or even millennia-old Ayurveda (Revathy et al., 2012; Seethapathy et al., 2019) and Asian traditional medicine systems (Masada, 2016; Xu et al., 2019). The substantial proportion of adulterated commercial herbal products described appears to be independent of the methods used for their analysis, traditional pharmacopoeial methods being employed, such as macroscopic inspection (van der Valk et al., 2017), microscopy (Ichim et al., 2020), chemical techniques (Li et al., 2008; Upton et al., 2020), or even the more recently developed DNA-based ones, such as the rapidly technologically evolving DNA barcoding and metabarcoding (Ichim, 2019; Grazina et al., 2020).

On the global market, herbal products are sold in an extremely diverse variety of forms, from single ingredient, unprocessed, raw, whole plants to multi-species, highly processed extracts. Therefore, the successful authentication of commercial herbal products reported by peer reviewed studies are a valuable and useful source of information which provide the necessary practicalities, including their strengths and the limitations, of employing the right methods for a specific type of product along the length of its value chain (Booker et al., 2012). Such analyses of peer-reviewed authentication reports focused exclusively on commercial herbal products have concluded that, microscopy, a traditional pharmacopoeial identification method, is cost-efficient and can cope with mixtures and impurities but it has limited applicability for highly processed commercial samples e.g. extracts (Ichim et al., 2020). On the other hand, DNA-based identification, only recently adopted by the first two national Pharmacopoeias (Pharmacopoeia Committee of P. R. China, 2015; British Pharmacopoeia Commission, 2018), facilitate simultaneous multi-taxa identification by using the DNA of different origins extracted from complex mixtures and matrices but false-negatives can be expected if the DNA has been degraded or lost during post-harvest processing or manufacturing (Raclariu et al., 2018a; Ichim, 2019; Grazina et al., 2020). In this respect, our review adds the much needed peer-reviewed, systematically searched information, about the successful use of chemical identification for the authentication of commercial herbal products. While doing so, our review also provides some missing pieces of the commercial herbal products’ authenticity puzzle.

## Methods

### Databases

#### Search Strategy

Four databases were systematically searched for peer reviewed records following the PRISMA guidelines (Moher et al., 2009) using combinations of relevant keywords, Boolean operators and wildcards: [(“herbal product” OR “herbal medicine” OR “traditional medicine” OR “food supplement” OR “dietary supplement” OR “herbal supplement” OR nutraceutical) AND (authentic* OR contaminat* OR substitut*)] for Web of Science, PubMed, Scopus, and [(“herbal product” OR “herbal medicine” OR “food supplement” OR “dietary supplement” OR “herbal supplement” OR nutraceutical) AND (authentication OR contamination OR substitution)] for ScienceDirect. The option “search alert” was activated for all four databases, to receive weekly updates after the literature search was performed. Furthermore, we used cross-referencing to identify additional peer-reviewed publications.

#### Selection Process and Criteria

*Identification*: 10,497 records were identified through database searching (WoS = 1,317, PubMed = 3,253, Scopus = 5,446, and ScienceDirect = 481), and 196 additional records from cross-referencing and the weekly updates from the four databases. Screening: after the duplicates had been removed, 2,326 records were collected and their abstracts screened. After screening, 1,745 records were excluded for not reporting data relevant for the chemical authentication of herbal products. Eligibility: 581 full-text articles were assessed and screened based on the following eligibility criteria: 1) The reported products had to be “herbal products”; the full wide range of commercial names was searched for and accepted for being included in our analysis. 2) The analyzed products had to be “commercial”; keywords such as “purchased”, “bought”, were accepted. Our analysis excluded samples which were obtained “cost-free”, a “gift” or “donated” by a person, institution or company. 3) The products had to be clearly allocated to a “country” or “territory” (e.g., European Union). 4) The conclusion “authentic”/“adulterated” had to be drawn by the authors of the analyzed studies. 5) The products had to be analyzed with a “chemical” method or techniques.

The set of retrieved full-text articles was further reduced by 446 that did not meet all eligibility criteria. Included: 135 records.

## Results

Different chemical methods have been successfully employed for the botanical authentication of 2,386 commercial herbal products, sold in 37 countries spread on six continents. The majority of the analyzed products were reported to be authentic (73%) but more than a quarter proved to be adulterated (27%), when the botanical identity of their content was compared with the label stated ingredients (Table 1).

**TABLE 1:** The authenticity of the chemically authenticated commercial herbal products at global level.

No. crt.	Country / territory	Products (details) / authenticated species	Products			Adulteration reported	Authentication method / marker (if reported)	Additional quality issues detected	Botanical/ chemical reference materials/ standards	Bibliographic reference
			total	authentic/ adulterated						
			no.	no.	no.					
1	Australia	grape seed extract products (capsules) from retail pharmacies, health stores / *Vitis vinifera*	9	4	5	complete substitution or heavy adulteration, possibly with peanut skin extract, *Pinus massoniana* (or other A-type procyanidin-containing species)	RP HPLC-UV-MS / catechin, epicatechin, procyanidin B2, procyanidin A2, rape seed oligomeric proanthocyanidins	not reported	*V. vinifera* (seeds, seed extracts), *A. hypogaea, P. massoniana, P. pinaster, V. macrocarpon, T. cacao* (extracts)	Govindaraghavan (2019)
	New Zeeland		6	6	0	n/a				
2	Australia	gingko products (capsule, tablets) from retail stores / *Ginkgo biloba*	6	3	3	adulteration with flavonol aglycones, likely with *Styphnolobium japonicum*	RP HPLC, LC-MS / flavonol aglycones (quercetin, kaempferol, isorhamnetin)	contained genistein, an isoflavone that does not occur in ginkgo leaf	authenticated samples of dried *Ginkgo biloba* leaf from commercial suppliers	Wohlmuth et al. (2014)
	Denmark		2	2	0	n/a				
3	Belgium	products (tablets and capsules) containing regulated plants / *Aristolochia fangchi*, *Ilex paraguariensis, Epimedium* spp., *Pausinystalia johimbe, Tribulus terrestris*	69	48	21	adulteration/ contamination with unlabeled ingredients: *A. fangchi* (forbidden), *I. paraguariensis, Epimedium* spp., *T. terrestris* (all should be notified to authorities), *P. johimbe*	FT-Mid-IR, HPLC-DAD, LC-MS	*P. yohimbe* or *T. terrestris* not identified in some products although claimed on the label	reference material of the five plant species (leaves, bark, fruits)	Deconinck et al. (2019)
4	Belgium	herbal products (capsules, tablets) from local pharmacy / *Passiflora edulis*	3	3	0	n/a	HPLC-DAD, HPLC-MS	not reported	commercial *P. edulis* (dry extract) (European Pharmacopoeia)	Deconinck et al. (2015)
5	Belgium	products containing three non-regulated herbs (capsule, tablets) from local pharmacy / *Frangula purshiana, Passiflora edulis, Crataegus monogyna*	3	3	0	n/a	HPLC-DAD–ELSD, HPLC-MS	not reported	commercial dry plant extracts of *F. purshiana, P. edulis, C*. *monogyna* (European Pharmacopoeia)	Deconinck et al. (2013)
6	Belgium	illegal products (tablets, capsules) containing regulated plant species / *Epimedium* spp., *Tribulus terrestris*	2	2	0	n/a	HPLC-PDA. HPLC-MS	adulteration with sildenafil	self-made triturations in three different botanical matrices from reference standards of *Epimedium* spp. leaves, *P. johimbe* bark, *T. terrestris* fruit	Custers et al. (2017)
7	Brazil	"carqueja" products (bags with pulverized plant material or parts of the plant) from commercial shops / *Baccharis trimera*	15	11	4	non-authentic	GC-FID / essential oil	intensity of the peaks in most of cases was different	authenticated samples of *B. trimera* (aerial parts, leaves) / standard oil of *B. trimera* (extracted)	De Ferrante et al. (2007)
8	Brazil	"sarsaparilla" products from drugstores / *Smilax goyazana, S. rufescens, S. brasiliensis, S. campestris, S. cissoides, S. fluminensis, S. oblongifolia, S. polyantha*	15	0	15	different from the reference *Smillax* sp.	TLC / flavonoids, saponins, terpenoids, steroids, catechins	n/a	authenticated reference material (roots) of *S. brasiliensis, S. campestris, S. cissoides, S. fluminensis, S. goyazana, S. oblongifolia, S. rufescens, S. polyantha*	Martins et al. (2014)
9	Brazil	"copaiba" oil-resin products from local markets / *Copaifera multijuga*	12	3	9	substitution and adulteration with soybean oil	TLC	not reported	reference *C. multijuga* oil-resins, prepared mixtures of soybean oil and copaiba oil resin	Barbosa et al. (2009)
10	Brazil	"carqueja" products from herbal shops, pharmacies / *Baccharis trimera*	12	12	0	n/a	TLC / 3-o-methyl-quercetin	large variations in the percentage of flavonoids (quercetin)	*B. trimera* reference samples / Brazilian Pharmacopoeia (BP)	Beltrame et al. (2009)
11	Brazil	"janaguba" milk products from local market / *Himatanthus drasticus*	10	4	6	complete substitution or adulteration with *Hancornia speciosa*	TLC	not reported	authentic samples of “janaguba” latex, mango tree latex sample	Soares et al. (2016)
12	Brazil	"Bauhinia spp." products (ground dry leaves) from drugstores, local market / *Bauhinia forficata* ssp.	9	2	7	not containing claimed B. forficata	HPLC-UV/PDA, MCR-ALS/PCA	not reported	*B. forficata, B. f.* var. *longifolia* authenticated leaves	Ardila et al. (2015)
13	Brazil	“jatoba” sap products / *Hymenaea stigonocarpa, Hymenaea martiana*	6	0	6	probably achieved by a decoction of the stem bark or other sources	HPLC-MS / flavonoids, procyanidins	n/a	*H. stigonocarpa, H. martiana* authenticated sap and stem bark samples	De Souza Farias et al. (2017)
14	Brazil	herbal products from commercial shops / *Maytenus ilicifolia*	3	1	2	possible substitution with plants from the same family and/or contamination due to addition of similar other plants parts to the commercial one	FTIR, 1H NMR	not reported	*M. ilicifolia* control sample from the open market, in the selected natural form, recognized by ‘‘herbal trackers’’	Preto et al. (2013)
15	Brazil	herbal products (raw material) from different suppliers / *Echinodorus grandiflorus*	3	3	0	n/a	TLC / caffeic acid, isoorientin and swertiajaponin, o-hydroxycinnamic acid derivatives	variable quantity of some marker compounds	Brazilian Pharmacopoeia (BP) 5th edition	Dias et al. (2013)
16	Canada	*Smilax ornata*, organic *Sarsaparilla* root, *Hemidesmus indicus* products from online store / *Hemidesmus indicus*, *Periploca indicus*	3	0	3	adulteration with *Decalepis hamiltonii* and *Pteridium aquilinum*	1H-NMR/HCA	not reported	reference samples of known provenance of *P. aquilinum, Smilax aristolochiifolia, D. hamiltonii, H. indicus*	Kesanakurti et al. (2020)
17	China	"Tong-guanteng" products from medicine markets, drug stores / *Marsdenia tenacissima*	62	61	1	substitution with *Tinospora sinensis*	TLC, HPLC / tenacissoside H	TS-H contents (0.39-1.09%) larger than that regulated in the Chinese Pharmacopoeia (0.12%)	genuine *M. tenacissima* herb	Yu et al. (2018)
18	China	ginseng products (pills, bag, injections, capsules, tablets, powders, dripping pills) from drugstores / *Panax ginseng, P. quinquefolius, P. notoginseng*	40	38	2	*P. ginseng* products adulterated (weak chromatographic peaks, and several marker compounds were not detected)	LC–MS / ginsenosides	in few products markers for PG not detected, signals for PN (ginsenoside Rf) very weak	authenticated ginseng crude drug samples	Yang et al. (2016)
19	China	Pinelliae rhizoma products from herbal medicine markets / *Pinellia ternata*	39	12	27	substitution with *Pinellia pedatisecta*	HPLC-DAD, HPLC-MS, LC-MS / triglochinic acid	not reported	authenticated batches of Pinelliae rhizoma and Pinelliae pedatisectae rhizoma / extracted and purified triglochinic acid	Jing et al. (2019)
20	China	"Wuweizi" (Schisandrae Chinensis Fructus) and "Nan-wuweizi" (Schisandrae Sphenantherae Fructus) products from pharmaceutical manufacturers, pharmacies / *Schisandra chinensis, S. sphenanthera*	36	34	2	substitution with *S. aphenanthera*	LC-DAD-MS, TLC, HPLC / schisandrin, anwulignan	not reported	authenticated batches of batches of Wuweizi and Nan-wuweizi, reference crude drugs, in-house prepared mixtures	Jiang et al. (2016)
21	China	American or Asian ginseng root products from stores / *Panax ginseng, P. quinquefolius*	31	28	3	adulteration and substitution of wild with cultivated ginseng	1H NMR-PCA / sucrose, glucose, arginine, choline, 2-oxoglutarate, malate, ginsenosides	not reported	n/a	Zhao et al. (2015)
22	China	"Chaihu" (Bupleuri Radix) products from major herbal distribution centres / *Bupleurum chinense, B. scorzonerifolium*	31	20	11	substitution with *B. longiradiatum, B. bicaule, B. falcatum, B. marginatum* var. *stenophyllum*	HPLC-ELSD, HPTLC / saikosaponins	great variation in the content of the major saikosaponins	authenticated samples of *B. chinense, B. scorzonerifolium, B. falcatum, B. longiradiatum, B. bicaule, B. marginatum* var. *stenophyllum*	Tian et al. (2009)
23	China	red yeast rice (RYR) commercial raw materials from supplement manufacturers / *Monascus purpureus* - fermented rice	31	21	10	did not show the presence of any monacolins analyzed	UHPLC–DAD–QToF-MS / monacolins, citrinin	n/a	RYR authenticated samples	Avula et al. (2014)
	United States	RYR-containing products from online retailers / *Monascus purpureus* - fermented rice	14	14	0	n/a		large variations (20-40 fold) in quantity and quality of monacolin K		
24	China	Asian and American ginseng products from herbal markets, local drug stores / *Panax ginseng, P. quinquefolius*	31	23	8	adulteration with *P. ginseng*	UPLC/Q-TOF-MS / ginsenoside Rf, 24 (R)-pseudoginsenoside F11	not reported	self-prepared samples with different contents (spiking the Asian ginseng powder into the American ginseng powder)	Li et al. (2010)
	Canada		5	5	0	n/a				
	United States		4	4	0	n/a				
25	China	"Gou-Teng" batches of (Uncariae Rammulus Cum Uncis) from markets / *Uncaria macrophylla, U. hirsuta, U. sinensis, U. sessilifructus*	20	16	4	substitution with other *Uncaria* sp. or unlabelled mixtures with the five officially accepted *Uncaria* sp.	UPLC/Q-TOF MS / alkaloids	not reported	authenticated batches of five *Uncaria* sp. (stems with hooks) / isolated and identified alkaloids	Pan et al. (2020)
26	China	Chaenomelis Fructus (raw) products from manufacturers, herbal markets / *Chaenomeles speciosa*	20	19	1	the source plant is not *C. speciosa*	HPLC–DAD / quinic acid, malic acid, protocatechuic acid, shikimic acid, chlorogenic acid	the relative contents of each component may vary in some of the samples	n/a	Zhu et al. (2019)
27	China	"Beimu" (Fritillariae Bulbus) products from drugstores / *Fritillaria taipaiensis, F. unibracteata var. wabuensis, F. delavayi, F. unibracteata, F. przewalskii, F. cirrhosa, F. ussuriensis, F. thunbergii*	16	11	5	substitution or adulteration with unlabeled *F. ussuriensis*	UPLC-QTOF-MS / steroidal alkaloids	loss of specific features, possibly resulted from different processes of different manufacturers	authenticated batches of *Fritilaria* sp.	Liu et al. (2020)
28	China	Menispermi Rhizoma products (dried rhizomes, pills, capsules) from drug stores / *Menispermum dauricum*	16	15	1	counterfeit (most of the important marker alkaloids could not be detected)	UPLC-DAD-MS / alkaloids	discrepancies among the samples of different origins (the contents of the nine alkaloids varied greatly)	authenticated MR batches from various drug stores / separated and purified (from MR) alkaloids	Liu et al. (2013a)
29	China	batches of "Shuxiong" tablets from manufacturers, drugstores / *Panax notoginseng, Carthamus tinctorius, Ligusticum striatum*	12	12	0	n/a	UPLC/QDa-SIM / (saponins, quinochalcone C-glycosides, 16 O-glycoside, phenolic acid, pathalides	low content of some markers in a few products possibly caused by different preparation process or use of poor-quality drug materials	crude drug reference materials Notoginseng Radix et Rhizoma, Carthami Flos, Chuanxiong Rhizoma	Yao et al. (2016)
30	China	"Huangqi" (Radix Astragali) products from wholesale TCM markets, city pharmacies / *Astragalus prompiquus*	12	11	1	substitution with *Astragalus tongonlensis*	HPLC-UV / isoflavonoids	total isoflavonoids content varies considerably	n/a	Wu et al. (2005)
31	China	"ci-wu-jia" tea products (leaf, leaf powder) from local stores / *Eleutherococcus senticosus*	11	8	3	adulteration with green tea (*Camellia sinensis*)	UHPLC-UV-MS/MS / organic acid derivatives, flavonoids, triterpene saponins	not reported	*E. senticosus* leaf samples collected from China / in-house UNIFI library of *Eleutherococcus* genus and green tea extracts	Wang et al. (2019)
32	China	*Panax ginseng* and *P. quinquefolius* products (bolus, tea, tablet, drink) from local pharmacies / *P. ginseng, P. quinquefolius*	11	10	1	substitution or adulteration with *P. ginseng*	UHPLC-TOF/MS/ OPLS-DA / ginsenosides	n/a	34 white ginsengs, 23 red ginsengs, 30 *P. notoginseng* and 21 *P. quinquefolius* collected samples	Wu et al. (2020)
33	China	*Panax notoginseng* powder products from drug stores, CHM manufacturers / *P. notoginseng*	10	9	1	adulteration, possibly with flower material of *P. notoginseng*	UPLC/Qtof MS/ PCA / notoginsenosides, ginsenosides, 20S-ginsenoside Rh1, gypenoside XVII	not reported	authenticated *P. notoginseng* powder samples	Liu et al. (2015)
34	China	"Xihuangcao" (Isodonis lophanthoidis herba) from herbal markets / *Isodon lophanthoides*	9	7	2	substitution with *I. lophanthoides* var. *gerardianus*	HPTLC / 2α-O-β-D-glucoside-12-en-28-ursolic acid, 2α,19α-dihydroxy-12-en-28-ursolic acid, 2α-hydroxy-12-en-28-ursolic acid, ursolic acid	not reported	collected batches of *I. lophanthoides*	Lin et al. (2019)
35	China	*Panax ginseng* products from local drug stores / *P. ginseng*	8	5	3	substitution with *P. quinquefolius, Platycodon grandiflorus, Physochlaina infundibularis, Phytolacca acinosa*	FT-NIR	not reported	authenticated *P. ginseng* samples	Dong et al. (2020)
36	China	"Xihuangcao" products (tea bags) from retail stores / *Isodon lophanthoides, I. serra*	8	0	8	no *Isodon* sp. material, adulteration and substitution with unlabeled plant species	UPLC-ESI-QTOF-MS	n/a	authenticated *I. lophanthoides* and *I. serra* plant material / reference teas of many plant species	Wan et al. (2016)
37	China	gingko leaf product and health foods (tea, tablets, soft gels) from drug store, local stores / *Gingko biloba*	6	5	1	adulteration (the rutin content was uncharacteristically high)	HPLC(EIS)/MS / flavonol glycosides, terpene trilactones, flavonol aglycones, biflavones	not reported	*G. biloba* leaves collected from different habitats	Song et al. (2010)
38	China	St. John's Worth products (loose material) from herbal markets, pharmacies and producer's cultivation / *Hypericum perforatum*	5	5	0	n/a	HPTLC, 1H-NMR/PCA	low content of typical H.p. compounds apparently due to higher amount of woody material	authenticated *Hypericum* sp. samples	Scotti et al. (2019)
	Bulgaria		2	2	0					
	Greece		2	2	0					
	Chile		1	1	0					
	United Kingdom		1	1	0					
39	China	Aquilariae Lignum Resinatum (ALR) products from market / *Aquilaria sinensis*	3	0	3	little or different resin components	FT-IR, SD-IR, 2D-IR	not reported	standard ALR (the resin-rich wood of *A. sinensis*	Qu et al. (2016)
40	China	Aquilariae Lignum Resinatum (ALR) products from market / *Aquilaria sinensis*	3	0	3	Adulteration with other kind of wood (possibly *Gonystylus* spp.), and by adding cheap resin (e.g. rosin)	FT-IR, 2D–IR	n/a	reference *A. sinensis* samples, no-resin wood of *A. sinensis*, authentic ALR samples	Qu et al. (2017)
41	China	*Ophiocordyceps sinensis* products from TCM market / *O. sinensis*	2	1	1	substitution with lepidopteran larvae infected by *Metacordyceps taii*.	HPLC / cordycepin, adenosine and other nucleosides	not reported	authenticated *O. sinensis* specimens collected in Tibet	Wen et al. (2016)
42	Croatia	gingko products (GBEs, food supplements / capsules, tablets, powder) / *Ginkgo biloba*	10	8	2	substitution with *Sophora japonica* extracts	HPLC / quercetin/ kaempferol ratio, ginkgo flavone glycosides (quercetin, kaempferol, isorhamnetin)	not reported	n/a	Budeč et al. (2019)
43	Denmark	St. John's Worth products (tablets, capsules) from commercial suppliers / *Hypericum perforatum*	10	10	0	n/a	1H-NMR/PCA	considerable differences in the products composition (e.g. flavonoids), inter-product and inter-batch variation	n/a	Rasmussen et al. (2006)
44	Egypt	herbal products (teas) from market / chamomile, marjoram, licorice, fennel, dill, caraway, basil, lemon grass, anise, chicory, achillea, verbascum, hibiscus, vine	3	0	3	adulterated with other species, some labeled species missing those of the formula	GC-MS, HPLC / essential oil, polyphenols, flavonoids	some of the herbs used are exhausted	reference herbal teas prepared from herbs purchased from the market	Kamal et al. (2017)
45	Egypt	herbal products (tea) / chicory, marjoram, nettle and senna leaves, liquorices roots, celery fruits, calendula flowers and fennel, senna and chicory	2	2	0	n/a	HPLC, GC-MS / sennoside A, esculetin, scopoletin. volatile oil	not reported	prepared standard herbal mixtures	Abdel Kawy et al. (2012)
46	European Union	*Panax ginseng* products (herb, root extracts, stem/leaf extract, berry extract) (capsules, tablets) / *P. ginseng*	12	6	6	*P. ginseng* leaf or other plant parts, *P. quinquefolius* roots	HPTLC, HPLC / ginsenosides	not reported	bulk crude *P. ginseng* dried root samples, *P. ginseng* leaf and stem	Govindaraghavan (2017)
	Australia		4	1	3	*P. ginseng* leaf or other plant parts				
	China		1	0	1	leaf/stem				
47	European Union	food supplements containing ginkgo dry extract or ginkgo leaf (tablets, soft and hard capsules) from local community pharmacies / *Ginkgo biloba*	10	2	8	adulteration	HPLC-UV, LC-MS/MS / flavonoids and terpenes lactones (ginkgolides, bilobalide)	n/a	*G. biloba* herbal medicinal product (control)	Czigle et al. (2018)
	Greece		1	0	1					
48	India	"Asoka" raw herbal products from shops / *Saraca asoca*	25	3	22	substitution	1D/2D NMR/PCA	not reported	taxonomically authenticated samples of *S. asoca* (bark, flower, stem)	Urumarudappa et al. (2016)
49	India	Garcinia products (capsules, tablets) from pharmacies, internet / *Garcinia gummi-gutta, G. indica*	5	5	0	n/a	1H NMR / (−)-hydroxycitric acid, (−)-hydroxycitric acid lactone	large variation in the content of (-)-hydroxycitric acid; only one product contained quantifiable amounts of (−)-hydroxycitric acid lactone	authenticated BRM from eleven species of *Garcinia* L.	Seethapathy et al. (2018)
	Norway		1	1	0					
	Romania		1	1	0					
	Sweden		1	1	0					
	United States		2	2	0					
50	India	licorice products (raw material) from local shops / *Glycyrrhiza glabra, G. uralensis, G. inflata*	2	2	0	n/a	HPTLC, HPLC / 18β-glycyrrhizic acid	not reported	vouchered, botanically confirmed sample, raw materials (whole, chopped, or powdered) of licorice root / United States Pharmacopeia (USP)	Frommenwiler et al. (2017)
51	Italy	bilberry products (extracts) from different producers / *Vaccinium myrtillus*	71	65	6	adulteration with anthocyanins extracted from other berries (black mulberry, chokeberry, blackberry)	HPLC-DAD, FT-NIR/PCA / anthocyanins and the respective aglycones	the amount of anthocyanins in the bilberry extracts in the range 18–34%	refined and standardized dry extract from the bilberry fruit	Gardana et al. (2018)
52	Italy	cranberry products (extracts) from herbal shops, local markets / *Vaccinium macrocarpon*	24	5	19	misidentification of the raw material	HPLC-UV/Vis, Orbitrap LC-MS / anthocyanins	only one product complied the criteria of good preparation, respected their uniformity of dosage, and contained V. macrocarpon	European Pharmacopeia	Mannino et al. (2020)
53	Italy	cranberry products (extracts) from herbal shops, local markets / *Vaccinium macrocarpon*	10	4	6	adulteration with *Morus nigra* extract	UPLC-DAD-Orbitrap-MS-PCA / anthocyanin, epicatechin/catechin, procyanidin A2/total procyanidin, procyanidin/anthocyanin ratios	only one product provided the daily dose deemed effective for treating a urinary tract infection	fruits and extract of possible adulterants	Gardana et al. (2020)
54	Italy	sweet fenel pre-packaged teabags and instant tea products (freeze-dried powders) from local pharmacies, grocery stores / *Foeniculum vulgare*	5	5	0	n/a	GC–MS / constituents of volatile oil	possible presence of bitter fennel or, for the powdered material, the presence of other parts of fennel	commercial reference samples of fruits of *F. vulgare* / European Pharmacopoeia (1997) monograph	Bilia et al. (2002)
55	Italy	herbal product (liquid preparations containing four species) from herbalist shop / *Olea europaea, Crataegus rhipidophylla, Fumaria officinalis, Capsella bursa-pastoris*	2	0	2	adulteration with a root extract from a *Rauvolfia* sp. (indole alkaloids)	HPLC-DAD–MS, HPLC–MS, NMR	n/a	purchased herbal products and collected plant material	Karioti et al. (2014)
56	Italy	herbal product (liquid preparations containing five species) / *Olea europaea, Crataegus rhipidophylla, Fumaria officinalis, Capsella bursa-pastoris*	1	0	1	adulteration with an extract from a *Rauvolfia* sp (indole alkaloids)	HPLC-ESI-ITMS, NMR	n/a	n/a	Gallo et al. (2012)
57	Japan	bilberry products (extracts) from the marketplace (tablets, hard and soft gel caps) / *Vaccinium myrtillus*	20	20	0	n/a	LC-MS / anthocyanins	marked composition differences	*V. myrtillus* reference dry extract	Cassinese et al. (2007)
	United States		15	7	8	substitution with berries different from *V. myrtillus*				
	Italy		4	2	2					
	Malaysia		1	0	1					
58	Japan	herbal products (crude drug extracts) (soft capsules, hard capsules, sugarcoated tablets) from internet / *Poria sclerotium, Ophiopogonis* tuber, *Rheum emodi*	14	1	13	mislabeling, adulteration	HPLC-PDA / sennoside A, aloe-emodin, emodin, rhein, chrysophanol	illegal adulteration with sibutramine	authenticated rhubarb rhizome	Yoshida et al. (2015)
59	Japan	chasteberry extracts (granules, tablets, soft and hard capsules) purchased via internet / *Vitex agnus-castus*	11	8	3	adulteration, contaminated with *V. negundo*	HPLC-PCA, quantitative determination of chemical marker compounds / agnuside, casticin	poor formulation quality	reference standard of *V. agnus-castus* fruit dry extract	Sogame et al. (2019)
60	Japan	herbal products (tea bags, granules, tablets) containing senna stems / *Cassia alexandrina*	8	5	3	adulteration with senna leaves and midribs	TLC, HPLC / sennoside A, sennoside B	the amount of sennosides ranged from 0.2-11 mg	reference raw senna materials (stems, leaves)	Kojima et al. (2000)
61	Japan	Siberian ginseng products (capsules, teas) from internet / *Eleutherococcus senticosus*	4	3	1	substitution with *Panax ginseng.*	HPLC-DAD / eleutheroside B, eleutheroside E, isofraxidin	not reported	specimens of *E. senticosus*, *E. sessiliflorus* and congeneric species, crude drugs from markets / chemical standards isolated from an authenticated commercial SG sample	Zhu et al. (2011)
62	Malaysia	"Tongkat Ali" products from pharmacies, night markets, jamu shops, food courts, on-line stores / *Eurycoma longifolia*	46	20	26	substitution	HPLC, 2DE / protein marker (A), eurycomanone	the amount of the markers detected varies among the products	purified *E. longifolia* crude extract	Vejayan et al. (2018)
63	Malaysia	‘Tongkat Ali’ products (capsules, spherical tablets) from pharmacies, drug stores / *Eurycoma longifolia*	29	18	11	substitution	2DE / protein markers (A, B) (∼14kDa)	not reported	standardized *E. longifolia* root extracts	Vejayan et al. (2013)
64	Malaysia	"Tongkat Ali" products (capsules, tea, tablet) from retail shops / *Eurycoma longifolia*	7	3	4	substitution	HPLC-DAD / eurycomanone	none of the products met the officially required minimum concentration of eurycomanone	authenticated *E. longifolia* plant and five-year-old root sample	Abubakar et al. (2018)
65	Mexic	"Damiana" botanical products (extracts) from local markets / *Turnera diffusa*	6	3	3	substitution, adulteration	1H-NMR/PCA / hepatodamianol	differences in the chemical components	authenticated *T. diffusa* specimens / purified chemical reference standard (hepatodamianol)	Lucio-Gutiérrez et al. (2019)
66	Pakistan	crude drugs from local market / *Foeniculum vulgarae, Curcuma longa, Aloe vera, Plantago ovata, Zingiber officinale, Glycyrrhiza glabra*	6	6	0	n/a	TLC, spectrophotometry, FTIR / anethole, barbaloin, xylose, galactose, gingerol-1, gingerol-2, 6-gingerol, glycerrihitic acid, curcumin	all the samples of *Plantago ovata* do not comply with the pharmacopoeial standard	n/a	Fatima et al. (2020)
67	Pakistan	"guggul" gum resin product from herbal market / *Commiphora wightii*	1	0	1	adulteration with *Mangifera indica* gum	NMR	n/a	authenticated gum resin samples of *C. wightii* and *M. indica*	Ahmed et al. (2011)
68	Poland	chamomile samples (fragmented, granulated) from different manufacturers / *Matricaria chamomilla*	19	19	0	n/a	HPLC / phenolic acids (gallic, caffeic, syringic, p-coumaric, ferulic), flavonoids (rutin, myricetin, quercetin, kaempferol)	not reported	n/a	Viapiana et al. (2016)
69	Poland	ginkgo products (leaf extracts) (capsules, tablets) from local pharmacies, markets, online pharmacies / *Ginkgo biloba*	16	9	7	adulteration probably with *Sophora japonica* (fruit or flower extracts)	ATR-FTIR, iPLS-DA / rutin, quercetin, kaempferol	large amounts of quercetin and kaempferol	standardized (24/6) ginkgo extracts	Walkowiak et al. (2019)
70	Poland	herbal products containing sage ethanolic extract (capsules, tablets, ointments, tincture, finished product) / *Salvia officinalis*	6	5	1	substitution	TLC / rosmarinic acid	not reported	*S. officinalis* authenticated botanical extracts	Cieśla and Waksmundzka-Hajnos (2010)
71	Romania	St. John’s Wort products (herbal teas, capsules, tablets, extracts) from pharmacies, herbal shops, supermarkets, internet / *Hypericum perforatum*	50	34	16	substitution with other *Hypericum* sp. or did not contain *Hypericum* species in detectable amounts	TLC, HPLC-MS / rutin, hyperoside, hyperforin, hypericin	not reported	authenticated reference plant material of *H. elegans, H. maculatum, H. olympicum, H. patulum, H. perforatum, H. polyphyllum*	Raclariu et al. (2017)
	Slovakia		3	1	2					
	Turkey		2	1	1					
	Austria		2	2	0	n/a				
	Czech Republic		1	1	0					
	France		1	1	0					
	Germany		4	4	0					
	Italy		1	1	0					
	Netherlands		1	1	0					
	Poland		4	4	0					
	Spain		2	2	0					
	Sweden		1	1	0					
	United Kingdom		2	2	0					
72	Romania	*Echinacea* products (teas, capsules, tablets, extracts) from retail stores, e-commerce / *Echinacea purpurea, E. angustifolia, E. pallida*	34	30	4	substitution or adulteration with unlabeled *Echinacea* sp.	HPTLC / echinacoside, cynarin, cichoric acid, chlorogenic acid, caffeic acid, caftaric acid	products totally devoided of any Echinacea sp. material	reference botanical standards: *E. purpurea, E. angustifolia, E. pallida* (UPS)	Raclariu et al. (2018b)
	Czech Republic		2	0	2					
	Germany		3	0	3					
	Italy		1	0	1					
	Poland		2	1	1					
	Spain		2	0	2					
	Austria		1	1	0	n/a				
	France		1	1	0					
	Norway		4	4	0					
73	South Korea	*Panax ginseng* (decoctions, beverages, capsules, tablets), *Platycodon grandiflorus* (decoctions, beverages), *Codonopsis lanceolata* (decoctions, beverages), *Pueraria montana* var. *lobata* (beverages) from local markets / *P. ginseng, P. grandiflorum, C. lanceolata, P. montana* var. *lobata*	81	81	0	n/a	HPLC, UPLC–DAD–ESI-IT-TOF-MS / lobetyolin, ononin	not reported	raw plant material of *P. ginseng, P. grandiflorum, C. lanceolata, P. montana* var. *lobata*	Choi et al. (2018)
74	South Korea	"Malabar tamarind" products from local market / *Garcinia gummi-gutta*	11	11	0	n/a	HPLC / cyanidin-3-O-sambubioside, cyanidin-3-O-glucoside	not reported	collected fruit rinds of *G. gummi-gutta*, purchased *G. indica* fruit samples	Jamila et al. (2016)
75	Taiwan	"myrobalan" (Fructus Chebulae) products from local herbal markets / *Terminalia chebula, Terminalia chebula* var. *tomentella*	28	20	8	substitution *with T. chebula* var. *parviflora*	HPLC / tannin-related constituents	not reported	reference standards, including some isolated previously from *T. chebula*	Juang and Sheu (2005)
76	Taiwan	herbal materials of Fritillariae Thunbergii Bulbus from local markets / *Fritillaria thunbergii*	12	12	0	n/a	HPLC-UV / peimine, peiminine	product with low total content of peimine (not to be used clinically)	n/a	(Lin et al., 2015)
77	Taiwan	white ginseng products (radix sliced material, powder, capsules) / *Panax ginseng*	8	7	1	not composed of 6 years old ginseng radix only	1H-NMR/PCA/CA	not reported	authenticated, one to six year-old, fresh white ginseng radix (*P. ginseng*)	Lin et al. (2010)
78	Taiwan	5:1 concentrated extract products (prepared from dried roots) from different companies / *Scutellaria baicalensis*	6	6	0	n/a	HPLC / baicalin, baicalein	significant product-to-product and batch-to-batch variation of the marker compounds	n/a	Ye et al. (2004)
	China		4	4	0					
79	Thailand	white "Kwao Krua" products from Thai local markets, drugstores / *Pueraria candollei*	7	7	0	n/a	HPLC / isoflavone glycosides (puerarin, daidzin, genistin), isoflavones (daidzein, genistein)	not reported	authenticated *P. candollei*, *Mucuna macrocarpa*, *Butea superba* plant material, Kwao Krua crude drugs	Intharuksa et al. (2020)
80	Thailand	*Garcinia atroviridis* products (capsules) from market / *G. atroviridis*	5	4	1	substitution	CZE / hydroxycitric acid and hydroxycitric acid lactone	not reported	n/a	Muensritharam et al. (2008)
81	Thailand	"Ya dok khao" smoking cessation tea product from local market / *Cyanthillium cinereum*	1	1	0	n/a	HPTLC / triterpenoid compounds (ß-amyrin, taraxasterol, lupeol, betulin)	not reported	*C. cinereum, E. sonchifolia* collected samples, raw *C. cinereum* materials	Thongkhao et al. (2020)
82	Turkey	chamomile products (tea bags, bulk or packaged crude flowers) from food stores, bazaar / *Matricaria chamomilla*	16	5	11	adulteration (possibly with *Anthemis* spp., *Tanacetum* sp. and *Chrysanthemum* sp.)	HPLC, HPTLC - PCA, HCA / apigenin 7-O-glucoside	A7G content in different tea brands ranged from 0.43-0.80 mg/g	wild and cultivated varieties of chamomiles, chamomile-like flowers (*Anthemis* L., *Bellis* L., *Tanacetum* L., *Chrysanthemum* L.)	Guzelmeric et al. (2017)
83	Turkey	Ginkgo products (extracts) from local pharmacy, local markets / *Ginkgo biloba*	13	13	0	n/a	LC-MS, HPLC-DAD / ginkgolides, flavonoid aglycones	total flavonoids and ginkgolides higher in medicinal products, no or very little flavonoids in food supplements	chemical reference standards (ginkgolides A, B, C, J), quercetin, kaempferol, rutin (isolated), isorhamnetin (prepared by acidic hydrolysis)	Demirezer et al. (2014)
84	Turkey	"okaliptus" products (leaves, essential oils) from herbal shops / *Eucalyptus globulus*	13	0	13	substitution with *E. camaldulensis*	TLC / essential oils	n/a	*E. camaldulensis, E. globulus, E. grandis* reference plant material / essential oils extracted from the reference plant material	Tombul et al. (2012)
85	United Kingdom	turmeric products (capsules, tablets, soft gels, powder, extracts) from stores, internet / *Curcuma longa*	50	48	2	absence of *C. longa*	1H-NMR/ PCA, HPTLC / curcumin , piperine, (S)-ar-Turmerone	significant quality variation between samples	n/a	Chatzinasiou et al. (2019)
	Germany									
	United States									
86	United Kingdom	St John's Wort products (tablets, capsules, powder) from internet, pharmacies, stores / *Hypericum perforatum*	22	14	8	adulteration (possibly with other *Hypericum* sp. obtained from China or use of chemically distinct *H. perforatum* cultivars or chemotypes)	HPTLC, 1H-NMR/ PCA	significant compositional variation among commercial finished products, adulteration with food dyes	SJW registered and quantified products, SJW EP Reference Standard	Booker et al. (2018)
	United States		17	8	9					
	Germany		8	7	1					
87	United Kingdom	*Sedum roseum* products (root and rhizome powders) (hard capsules, soft gel capsules, tables) from retail outlets, internet / *S. roseum*	39	32	7	substitution, adulteration with other *Rhodiola* sp. (e.g. *R. crenulata*)	HPTLC, MS, 1H NMR / rosavin, salidroside	lower rosavin content, substitution with 5-hydroxytryptophan	*S. roseum* crude drug material, *R. crenulata* aqueous extracts	Booker et al. (2016b)
88	United Kingdom	Ginkgo food supplements (tablets, hard capsules, caplets) from health food stores, supermarkets, pharmacies, internet/ *Ginkgo biloba*	33	5	28	adulteration (not in compliance with their label specification)	1H NMR/ PCA, HPTLC / flavonoids, terpene lactones	variable quality (different from that described in pharmacopoeias)	quantified and licensed Ginkgo extracts, *G. biloba* leaf samples	Booker et al. (2016a)
89	United Kingdom	American ginseng, white Asian ginseng, sanchi ginseng samples from importing companies / *Panax ginseng, P. quinquefolius, P. notoginseng*	8	8	0	n/a	LC/MS/MS / malonyl-ginsenosides	not reported	authentic root samples of *P. ginseng, P. quinquefolius, P. notoginseng*	Kite et al. (2003)
90	United Kingdom	herbal tinctures from health shop / *Echinacea purpurea, Hypericum perforatum, Ginkgo biloba, Valeriana officinalis*	4	4	0	n/a	1H-NMR, MS / hyperforin, hypericin, ginkgolic acids, terpene lactones ginkgolides A, B, and C	not reported	n/a	Politi et al. (2009)
91	United Kingdom	herbal product (capsules) / *Equisetum arvense*	3	1	2	no *Equisetum* sp. material (no TLC chromatogram)	TLC / kaempferol glucosides	not reported	material deposited in herbarium / characters used in the European Pharmacopoeia to identify *Equisetum* sp.	Saslis-Lagoudakis et al. (2015)
	Bulgaria	herbal product (tea) / *E. arvense*	1	0	1	adulterated with *E. palustre*				
	Germany	herbal product (tea) / *E. arvense*	1	1	0	n/a				
92	United States	bitter orange products (tablets, capsules, gel-containing capsules, drink powders) from online / *Citrus aurantium*	59	59	0	n/a	LC–MS/MS / phenethylamines (synephrine, octopamine, tyramine, N-methyltyramine, hordenine)	very few appear to meet claims for their label concentration declarations	n/a	Pawar et al. (2020)
93	United States	*Echinacea* preparations (tablet, caplet, capsule, liquid, powder, granule) from health food, drug, and grocery stores / *E. purpurea, E. angustifolia, E. pallida*	49	31	18	adulteration, substitution with unlabeled *Echinacea* sp., no measurable *Echinacea*	TLC / cichoric acid, echinacoside	variability in chemical composition	n/a	Gilroy et al. (2003)
94	United States	herbal supplements (loose powders, capsules, tablets, liquid extracts, dried fruit forms) to contain cranberry, lingonberry, bilberry, or blueberry from local stores or internet / *Vaccinium macrocarpon, V. vitis-idaea, V. myrtillus, V. corymbosum*	41	27	14	adulteration and substitution with *Vaccinium* sp.	HPLC/DAD / anthocyanins (cyanidin-3-glucoside)	wide variation of the anthocyanin content	verified authentic fruit with known anthocyanin profiles, anthocyanin profiles of small authenticated fruit samples	Lee (2016)
95	United States	goldenseal products (dried material, extract, freeze-dried material) (capsules, tinctures, powdered bulk materials, tea bags) from online / *Hydrastis canadensis*	35	32	3	adulteration with *Berberis. vulgaris, B. aquifolium, Coptis. chinensis*	LC-MS/PCA / berberine, hydrastine, canadine	not reported	reference materials (*H. canadensis, C. chinensis, B. aquifolium, B. vulgaris*) / canadine reference (isolated and purified from *H. canadensis*)	Wallace et al. (2018)
96	United States	black cohosh products (powder, dried extract, liquid extract) (capsules, tablets, soft gels, drops) from local stores or Internet / *Actaea racemosa*	33	19	14	not containing *A. racemosa* material	UPLC-PDA, UPLC-MRM / V9c and V9a markers, caffeic acid, ferulic acid, isoferulic acid	not containing the full spectrum of plant chemicals after preparation process	authenticated rhizome/root materials from different *Actaea* sp.	Geng et al. (2019)
97	United States	ginkgo products (tablets, capsules, caplet) from health food stores, supermarkets / *Ginkgo biloba*	27	27	0	n/a	HPLC / flavone glycosides, terpene lactones, ginkgolic acids	relevant compositional differences, particularly with regard to the content of ginkgolic acids	EGb 761 extract	Kressmann et al. (2002)
98	United States	"‘buchu" products (whole leaves, powders, capsules, tea bag) / *Agathosma betulina*	27	16	11	not containing labeled *A. betulina* or *A. crenulata*	HPTLC / rutin, chlorogenic acid, kaempferol	not reported	*A. betulina, A. crenulata* plant reference material	Raman et al. (2015)
99	United States	yohimbe products (powder, caplet, capsules, liquid, powdered drink mix) from retail health food outlets / *Pausinystalia johimbe*	26	17	9	not containing yohimbe material	GC/MS / yohimbine HCl, ajmaline, corynanthine	containing only trace amounts of yohimbine, largely devoid of the other alkaloids, possible presence of undeclared diluents	authenticated johimbe bark	Betz et al. (1995)
100	United States	ginseng preparations from the genera *Panax* or *Eleutherococcus* from local health food store / *P. ginseng, P. quinquefolius, P. notoginseng, E. senticosus*	25	25	0	n/a	LC-MS, HPLC / ginsenoside (Rb1, Rb2, Rc, Rd, Re, Rf, Rg1), eleutheroside (B and E)	product-to-product variability in the amount of ginsenosides or eleutherosides present	n/a	Harkey et al. (2001)
101	United States	German chamomile, Roman chamomile and Juhua products (crude drugs, capsules, tea bags, crude drugs mixed with other plant materials, powder, extracts) from supermarkets, local retail pharmacies, online / *Matricaria chamomilla, Chamaemelum nobile, Chrysanthemum morifolium*	24	20	4	substitution (not containing the labeled chamomille species) did not contain any detectable volatile components	GC/MS, PLS-DA / volatile compounds (b-Farnesene, a-bisabolol oxide A, B)	not reported	authenticated *C. nobile, M. chamomilla, C. morifolium* samples / essential oil samples obtained from the authenticated plant materials	Wang et al. (2014a)
	China		11	11	0	n/a				
102	United States	grape seed powder products (capsules) from vitamin supplement retailers, supermarkets, online / *Vitis vinifera*	21	12	9	adulteration with peanut skin extract	HPLC/UV/MS, LC–MS, TLC / proanthocyanidin B-type dimers	wide degree of variability in chemical composition	authenticated grape seed extract, peanut skin extract, pine bark extract	Villani et al. (2015)
103	United States	gingko products (leaf extracts) from food supermarkets, local retail pharmacies, online / *Ginkgo biloba*	21	21	0	n/a	GC/MS, LC/MS, UHPLC/MS / ginkgolic acids, terpene trilactones, flavonol glycosides	not reported	*G. biloba* authenticated and commercial plant samples (leaves, seeds, leaf extracts, sarcotesta)	Wang et al. (2014b)
104	United States	American and Korean ginseng products (fresh or dried roots) (powders, capsules, tablets) from local and national herbal health care stores / *Panax ginseng, P. quinquefolius*	20	18	2	devoid of ginseng material	RP-HPLC / ginsenosides (Rf, Rb1, Rc)	not reported	n/a	Mihalov et al. (2000)
	China		2	2	0	n/a				
105	United States	black raspberry products (freeze-dried whole and pre-ground powders) (capsules, extract, liquid) form internet / *Rubus occidentalis*	19	12	7	possible substitution with blackberry (*Rubus* spp.)	HPLC/DAD/MS / anthocyanins (cyanidin-3-glucoside)	wide range of anthocyanin concentration	n/a	Lee (2014)
106	United States	milk thistle products (capsules with dried, oil-based extracts) from market / *Silybum marianum*	19	19	0	n/a	U-HPLC-HRMS / silymarin flavonoids, flavonolignans	marked differences in the content of individual flavonoids/flavonolignans, even within different batches by the same manufacturers	reference dried milk thistle extract	Fenclova et al. (2019)
	Czech Rep		7	7	0					
107	United States	black cohosh products (dry extracts, powdered plant material) (capsules, tablets) from pharmacies, internet / *Actaea racemosa*	19	7	12	subtitution and adulteration with *C. dhurica, C. foetida*	LC-MS/MS / actein, 23-epi-26-deoxyactein	not reported	Cimicifuga Rhizome (JP16) samples from different companies	Masada-Atsumi et al. (2014)
	Germany		5	5	0	n/a				
	Switzerland		1	1	0					
108	United States	*Aloe vera* products / *Aloe vera*	18	18	0	n/a	1H-NMR / nicotinamide	differences among products (possible deacetylation)	authenticated *A. vera* samples (inner leaf powder, decolorized whole leaf freezing dried powder), Aloe acetylated polysaccharides reference standard	Jiao et al. (2010)
109	United States	*Tinospora* products from internet (capsules, caplets, granule, powder) / *T. crispa, T. sinensis*	17	15	2	substitution with *T. sinensis*	UHPLC-PDA-MS / flavonoid, alkaloids, amid, diterpenoids	not reported	reference plant samples of *T. crispa, T.sinensis, T. baenzigeri*	Parveen et al. (2020)
110	United States	skullcap and Chinese skullcap based dietary supplements from internet / *Scutellaria lateriflora, S. baicalensis*	15	6	9	substitution with *S. baicalensis* or *Teucrium canadense*	FI/MS/PCA / baicalin, verbascoside	very low *S. lateriflora* concentration	authenticated samples of *S. lateriflora* (aerial parts)	Sun and Chen (2011)
111	United States	"guarana" products (dried seeds, dried paste, seed powders, tablets, capsule) from local health food outlets, manufacturers, internet / *Paullinia cupana*	14	7	7	substitution (devoid of *P. cupana* material)	LC / theobromine, theophylline, caffeine, catechin, epicatechin	possible fortification with synthetic caffeine and dilution with inert ingredients	authenticated guarana seeds, dried paste	Carlson and Thompson (1998)
112	United States	*Hoodia gordonii* products (gels, capsules, tablets, sprays, teas, snack bars, powders, juices) / *H. gordonii*	13	2	11	substitution (no *H. gordonii* detected, other botanicals present)	HPTLC / pregnane glycosides (hoodigosides, P57)	not reported	various *Hoodia* sp. / isolated chemical reference standards	Rumalla et al. (2008)
113	United States	saw palmetto products (soft and hard gel capsules, tablets, tinctures) from retail outlets, pharmacies / *Serenoa repens*	13	13	0	n/a	GC, 1H-NMR/PCA / quantification of fatty acids	inaccurate labeling of fatty acid content	n/a	Booker et al. (2014)
	United Kingdom		11	11	0					
	Canada		7	7	0					
	Netherlands		7	7	0					
	Switzerland		6	6	0					
	Spain		5	5	0					
	South Korea		4	4	0					
	Finland		1	1	0					
	Germany		1	1	0					
114	United States	St. John’s Wort (herb/aerial parts, extracts) products from market, online / *Hypericum perforatum*	12	6	6	adulteration (possible mixtures with *H. undulatum*)	HPTLC / rutin, hypericin, pseudohypericin	not reported	*H. perforatum* extract standard, *H. undulatum, H. montanum, H. tetrapterum*, and *H. hirsutum* samples	Frommenwiler et al. (2016)
115	United States	goldenseal products (capsules, raw, tea bag, liquid extract) from local retailers or internet / *Hydrastis canadensis*	12	12	0	n/a	HPLC / berberine chloride, (ÿ)-b-hydrastine	wide range of content variation for hydrastine (0.00–2.51%) and berberine (0.00–4.35%)	authenticated crude goldenseal powder	Abourashed and Khan (2001)
116	United States	"yohimbe" products (bark cut and sifted pieces, powders) from online / *Pausinystalia johimbe*	12	8	4	adulterated, yohimbine not detected	UPLC-UV-MS / yohimbine	products range widely in yohimbine content (0.1–0.91%)	authenticated *P. johimbe* bark samples	Raman et al. (2013)
117	United States	black cohosh products (extracts, powdered plant material) (tablets, capsules) from stores / *Actaea racemosa*	11	7	4	substitution and contamination with Asian *Actaea* species	TLC, HPLC, LC-MS / triterpene glycosides, phenolics	significant product-to-product variability in the amounts of the selected triterpene glycosides and phenolic constituents	authenticated plant material of *Actaea cimicifuga, Actaea dahurica, Actaea yunnanensis*	Jiang et al. (2006)
118	United States	pure *Hoodia gordonii* producs from the market / *H. gordonii*	10	1	9	substitution with *H. parviflora*, contamination	1H NMR / P57, hoodigoside L	not reported	authenticated samples of *H. gordonii, H. parviflora, H. ruschii, H. currorii* / isolated chemical reference standards	Zhao et al. (2011)
119	United States	goldenseal products (root/rhizome) (capsules) from internet / *Hydrastis canadensis*	10	10	0	n/a	LC-UV, LC-MS / berberine, canadine, hydrastine, coptisine, palmatine, jatrorrhizine, dihydrocoptisine	not reported	reference samples (dried powders) of *H. canadensis* (root), *Coptis chinensis* (root)	Wallace et al. (2020)
120	United States	cranberry products (powders, concentrate, fruit solids) from common vendors or internet / *Vaccinium macrocarpon*	9	3	6	adulteration (with extracts from other plant species)	1H-NMR / triterpenoids, organic acids, total proanthocyanidins and anthocyanins	substantially variation of the metabolic profile, slightly lower PAC content may be caused by removal during manufacturing	*V. macrocarpon* freeze dried fruit powder, whole cranberry fruits of different cultivars	Turbitt et al. (2020)
121	United States	"ma-huang" products from local retailers, internet / *Ephedra sinica*	9	9	0	n/a	HPLC / ephedrine-type alkaloids	considerable variability in alkaloid content (EPH 1.08–13.54 mg) and lot-to-lot variations in EPH of 137%.	unprocessed *E. lematolepis*	Gurley (1998)
122	United States	standardized (24/6) ginkgo products (leaf extracts) from suppliers / *Ginkgo biloba*	8	5	3	adulteration (possibly with sophora extracts)	HPLC-DAD / flavone glycosides	high levels of quercetin and kaempferol	certified ginkgo extract 24/6, commercial extracts of *Styphnolobium japonicum*	Chandra et al. (2011)
123	United States	*Vangueria agrestis* products (extracts) / *V. agrestis*	7	4	3	adulteration	HPTLC / saponins, flavonoids, phenolics, iridoid	not reported	authenticated *V. agrestis* samples (twigs with intact leaves, stems, roots)	Raman et al. (2018)
124	United States	American ginseng products from supermarkets / *Panax quinquefolius*	6	4	2	substitution with *P. ginseng*	HPLC/HCA/PCA / ginseng saponins	not reported	standard *P. ginseng, P. notoginseng* samples, *P. quinquefolius* samples from USA, Canada, China	Yu et al. (2014)
125	United States	African mango products from internet / *Irvingia gabonensis*	5	1	4	substitution (do not contain detectable amount of authentic material)	UHPLC-PDA-HRMS / ellagic acid, mono-, di-, tri-O-methyl-ellagic acids and their glycosides	trace constituents of regular mango seeds	*M. indica* samples	Sun and Chen (2012)
126	United States	*Echinacea* products (tablets, capsules, powder) / *Echinacea purpurea*	5	1	4	adulteration	HPLC-CAD	not reported	*Echinacea* sp. (extracts, root, herb)	Waidyanatha et al. (2020)
127	United States	plantain products (tablets) / *Plantago major*	5	4	1	contamination with *Digitalis lanata*	Kedde reaction, TLC, LC-MS / cardiac glycosides (lanatosides A, B, C, digoxin, digitoxin)	not reported	n/a	Slifman et al. (1998)
128	United States	black cohosh products from health store, marketplace / *Actaea racemosa*	4	3	1	substitution with *Cimicifuga foetida*	HPLC-PDA/MS/ELSD / (triterpene glycosides, phenolic compounds)	product inadequately manufactured (overheating)	*Actaea* sp. plant material / authentic *Cimicifuga* chemical reference standards	He et al. (2006)
129	United States	passion flower products (capsules) from online / *Passiflora edulis*	4	4	0	n/a	UPLC-UV-MS, HPTLC / flavonoids, harmane-carboline alkaloids	not reported	authenticated aerial parts of *P. edulis, P. violacea, P. suberosa, P. morifolia, P. quadrangularis*, seeds of *Peganum harmala*	Avula et al. (2012)
130	United States	feverfew extracts (capsules, drops) / *Tanacetum parthenium*	3	3	0	n/a	LC-UV/LC-MS / parthenolide	not reported	*T. parthenium* and *T. vulgare* plant material	Avula et al. (2006)
131	United States	herbal products (tea, capsules) / *Equisetum arvense*	3	3	0	n/a	TLC / kaempferol glucosides	not reported	material deposited in herbarium / characters used in the European Pharmacopoeia to identify *Equisetum* sp.	Saslis-Lagoudakis et al. (2015)
132	United States	goldenseal products (root powder) from bulk suppliers / *Hydrastis canadensis*	3	2	1	adulteration, possibly with *Coptis* root or barberry bark	LC-MS / alkaloids (berberine, hydrastine, canadine)	not reported	*Coptis japonica* root powder, *Berberis aquifolium* root powder, *Chelidonium majus* herb, *Berberis vulgaris* bark powder	Weber et al. (2003)
133	United States	ginseng products (liquid extract, capsules) from a local nutritional store / *Panax quinquefolius, P. ginseng, P. notoginseng*	2	2	0	n/a	UPLC/QTOF-MS/PCA / (ginsenosides, pseudoginsenosides, gypenosides, notoginsenosides)	not reported	authenticated ginseng roots (*P. quinquefolius, P. ginseng, P. notoginseng*)	Yuk et al. (2016)
134	United States	African mango sample (powdered seeds) / *Irvingia gabonensis*	1	0	1	contamination or adulteration with goji berry (*Lycium barbarum*)	HPLC-PDA, LC-IT-MS, 1H NMR / pyrrole alkaloid	n/a	authentic sample of African mango seed powder, goji berries	Li et al. (2014)
135	United States	American skullcap (freeze-dried) product / *Scutellaria lateriflora*	1	1	0	n/a	HPLC / flavonoids (baicalin, baicalein, wogonin)	not reported	*S. lateriflora* (aerial parts) reference material	Brock et al. (2013)
	**Total**		**2,386**	**1,734**	**652**					

The herbal products were purchased from 37 countries scattered over six continents: Europe (*n* = 20), Asia (*n* = 9), North America (*n* = 3), Australia (*n* = 2), South America (*n* = 2), and Africa (*n* = 1) (Supplementary Table S1). The numbers of reported samples were geographically heterogeneous, at continental level the highest number of commercial herbal products was reported for Asia (*n* = 877), North America (*n* = 767), Europe (*n* = 573), followed distantly by South America (*n* = 86), Australia (*n* = 25) and Africa (*n* = 5). The proportion of adulterated products varies significantly among continents, being highest in Africa (60%), South America (57%), Australia (44%), and lower in Europe (28%), North America (27%), and Asia (25%). The adulteration percentage of the last three continents enumerated is close to the global one (27%) which can be influenced also by the significantly higher number of commercial products analyzed and reported, compared with the samples analyzed from the other three continents.

The distribution of commercial samples among the 37 countries is highly heterogeneous as well (Table 2). More than 100 commercial products were reported for four countries, i.e. United States (*n* = 746), China (*n* = 491) followed distantly by United Kingdom (*n* = 123) and Italy (*n* = 119). Another seventeen countries are well represented (*n* ≥ 10) by the successfully analyzed samples, while the other sixteen countries have even fewer (*n* < 10) products reported.

**TABLE 2:** The distribution and authenticity of the chemically authenticated commercial herbal products at national level.

Country/Territory	Products	Authentic products		Adulterated products	
	no.	no.	%^a^	no.	%^b^
United States	746	548	73	198	27
China	491	388	79	103	21
United Kingdom	123	78	63	45	37
Italy	119	82	69	37	31
South Korea	96	96	100	0	0
Brazil	85	36	42	49	58
Romania	85	65	76	20	24
Malaysia	83	41	49	42	51
Belgium	77	56	73	21	27
Japan	57	37	65	20	35
Taiwan	54	45	83	9	17
Poland	47	38	81	9	19
Turkey	44	19	43	25	57
India	32	10	31	22	69
Germany	22	18	82	4	18
European Union^b^	22	8	36	14	64
Australia	19	8	42	11	58
Canada	15	12	80	3	20
Thailand	13	12	92	1	8
Denmark	12	12	100	0	0
Croatia	10	8	80	2	20
Czech Republic	10	8	80	2	20
Spain	9	7	78	2	22
Netherlands	8	8	100	0	0
Pakistan	7	6	86	1	14
Switzerland	7	7	100	0	0
Mexico	6	3	50	3	50
New Zeeland	6	6	100	0	0
Egypt	5	2	40	3	60
Norway	5	5	100	0	0
Austria	3	3	100	0	0
Bulgaria	3	2	67	1	33
Greece	3	2	67	1	33
Slovakia	3	1	33	2	67
France	2	2	100	0	0
Sweden	2	2	100	0	0
Chile	1	1	100	0	0
Finland	1	1	100	0	0

^a^ The percentage values were rounded to the nearest whole number.

^b^ Not reported by the authors the exact EU country.

In twelve countries, out of the total of thirty-seven, all the analyzed commercial herbal products (100%) were reported as authentic, albeit, for eight of them, less than 10 samples were reported. Notably, the botanical identity of the samples purchased from South Korea (*n* = 96) and Denmark (*n* = 12) matched the labeled information. The adulterated proportion in the remaining twenty-five countries varied widely, from 8% up to as much as 80%. From the countries where more than 10 samples from their marketplace have been chemically authenticated and non-authenticated products have been reported, the majority of the commercial products was adulterated, being the highest in India (69%), followed closely by Australia (58%), Brazil (58%), Turkey (57%) and Malaysia (51%). Noticeably, the adulteration percentage of the four countries with more than 100 commercial products reported is 37% (United Kingdom), 31% (Italy), 27% (United States) and the lowest is reported for China (21%).

### Sampling Heterogeneity and Unavoidable Bias

The authentication raw data were all retrieved from peer-reviewed articles, the vast majority of them after they were indexed in the four major international databases which were systematically searched for while some other few articles were identified after cross-referencing. Although no limiting criteria (e.g. publication year, or language) was used, the authentication data reported in journals with limited-impact and international visibility might be underrepresented in the retrieved data. Moreover, the access of researchers from the economically depressed economies to high-impact journals, and especially to the OA journals, is a further limiting factor for publicly communicating the authentication results relevant for a certain country. On the other hand, as it was previously mentioned as possible bias, also the countries with a functional consumer safety system might be underrepresented as the authentication results of the commercial samples screened by the respective institutions will be published in internal bulletins or protocols, rather than in peer-reviewed journals (Ichim et al., 2020).

## Discussion

The chemical identification methods have confirmed that a substantial proportion (27%) of herbal products from the international market place is adulterated: on average, more than one in each four products sold in the 37 countries included in our analysis was proved to be non-authentic regarding their botanical identity. This adulteration percentage, revealed by employing many and very diverse chemical analytical methods, almost matches the figure obtained after the use of DNA-based techniques were assessed for their use for the authentication of commercial herbal products in a comparable number of countries: 27% (Ichim, 2019). Indeed, this percentage was obtained after almost a triple number of commercial herbal products (*n* = 5,957) were analyzed and their results reviewed recently. Notably, the microscopic authentication of commercial herbal products have reported a much higher adulteration rate (41%) but the number of analyzed samples was considerably much smaller (*n* = 508) which can be a possible bias of this finding (Ichim et al., 2020).

As it was previously reported by many peer-reviewed reports (Hoban et al., 2018; Seethapathy et al., 2019; Amritha et al., 2020; Anthoons et al., 2021; Palhares et al., 2021), irrespective of the authentication method, adulterated commercial HPs are geographically present across all continents (Supplementary Table S1). Moreover, this highly relevant category of commercial products was found to not comply with the labeled botanical ingredients in proportions almost identical (26 ± 2%), irrespective if they are traditionally used as herbal medicines, as commonly found in Asia, or overwhelmingly consumed as food supplements as in Europe or North America. These two main categories of herbal products commercialized in the global marketplace have many types of value chains (Booker et al., 2012), with some different stakeholders and entities along their shorter or more complex trade chains. Nevertheless, the end-users of both systems seem to be equally affected by non-authentic, accidental contamination or fraudulent substitution of labeled botanical ingredients and even the addition of compounds in an attempt to fool quality control testing e.g. as in adding food dyes to *H. perforatum* in order to achieve higher UV spectroscopy readings (Booker et al., 2018). Indeed, although monographs for herbal raw materials (e.g., Ph. Eur, USP) allow a minor presence of foreign organic matter (Parveen et al., 2016), the adulteration patters documented by employing different chemical methods, are very diverse and most of them are made possible only by the intentional, economically motivated and fraudulent actions of onerous producers or traders.

The total absence of labeled botanical ingredients and/or their extracts from the commercial herbal products tested was detected by using chemical methods. Commercial samples devoid of labeled botanical ingredient species (Carlson and Thompson, 1998; Ardila et al., 2015; Geng et al., 2019; Zhu et al., 2019) or not even substituted with their related species (Wan et al., 2016). An easy way to increase the profit margin of the products was the use of cheaper plant material as it was the use of other plant parts than the ones recommended, labeled and expected by the product’s users, senna (*Senna alexandrina* Mill.) stems substituted with leaves and midribs (Kojima et al., 2000), *Panax ginseng* C.A.Mey roots with other plant parts (leaf or stem) (Govindaraghavan, 2017), or *Panax notoginseng* Burkill F.H.Chen roots with flowers (Liu et al., 2015). Another similar deceptive adulteration strategy was the reported use of extracts obtained from plant parts other than the recommended ones, such as the decoction of the stem bark to substitute the genuine “jatoba” sap products (*Hymenaea stigonocarpa* Hayne*, Hymenaea martiana* Hayne) and the adulteration of Aquilariae Lignum Resinatum (*Aquilaria sinensis* (Lour.) Spreng) products with cheap resin (e.g. rosin) (Qu et al., 2017). The economically motivated adulteration includes also the use of unlabeled filler species as the DNA of species such as rice (*Oryza sativa* L.), soybean (*Glycine max* (L.) Merr.) and wheat (*Triticum* spp.) was previously identified in commercial herbal products (Newmaster et al., 2013; Ivanova et al., 2016). Yet, the TLC alone was able to detect the fraudulent use of soybean oil as filler in “copaiba” (*Copaifera multijuga* Hayne) oil-resin products (Barbosa et al., 2009).

The detection of unlabeled species with allergenic potential and known or suspected toxicity was previously reported by the use of DNA-based authentication techniques (Newmaster et al., 2013; Speranskaya et al., 2018). The same potential was shown by the phytochemical analyses which have been able to unmask the presence of unwanted and hazardous botanic ingredients, such as species that should have been notified to authorities (e.g. *Ilex paraguariensis* A. St-Hil., *Epimedium* spp., *Tribulus terrestris* L*.*), or forbidden toxic plants (e.g. *Aristolochia fangchi* Y.C.Wu exL.D.Chow and S.M.Hwang) (Deconinck et al., 2019) or even health hazardous contaminations, with *Digitalis lanata* Ehrh. added to plantain (*Plantago major* L*.*) products (Slifman et al., 1998). Moreover, as peanut allergy is a major public health concern and can be severe or even life-threatening (Gray, 2020), chemical methods have proved able to detect adulteration with the peanut skin extract of grape seed-containing herbal products (*Vitis vinifera* L.) from Australia (Govindaraghavan, 2019) and United States (Villani et al., 2015).

All the intentional adulteration practices documented and reported repeatedly till now (Li et al., 2008; Ichim, 2019; Xu et al., 2019; Ichim et al., 2020; Upton et al., 2020) can be evidenced by peer-reviewed reports referring to the top selling herbal products containing highly valued or widely used medicinal species across countries and cultures. The prices of ginseng herbal medicines and supplements vary widely based on the species, quality, and purity of the ginseng, and this provides a strong driver for intentional adulteration (Ichim and de Boer, 2021). Indeed, several chemical methods were able to identify ginseng products totally or partially devoid of the labeled *P. ginseng* plant material (Mihalov et al., 2000; Yang et al., 2016) and prove that, in most cases, labeled *Panax* species were substituted with other *Panax* species (Li et al., 2010; Yu et al., 2014; Dong et al., 2020), but also the substitution of ginseng root with leaves, stems or flowers (Liu et al., 2015; Govindaraghavan, 2017). Notably, chemical analysis was even able to detect the adulteration and substitution of wild with cultivated ginseng (Zhao et al., 2015) as well as a white ginseng products (*P. ginseng*) not composed of 6 years old ginseng radix only (Li et al., 2010).

Studies carried out at UCL School of Pharmacy, London have consistently shown that product adulteration is commonplace, with 25–40% of products typically being found to be of poor quality or adulterated, and especially with products obtained via the internet. Although with products that have been registered as Traditional Herbal Medicines under the Traditional Herbal Medicinal Products Directive (THMPD), no adulteration has so far been found and these products have shown to be of acceptable quality (Booker et al., 2016a; Booker et al., 2016b; Booker et al., 2018). This does not necessarily mean that all non-registered products (e.g. food supplements) are of poor quality but the problem being that it is difficult for the general public to be able to reliably discern high quality products from inferior ones. Organic certification provides some assurances regarding traceability, including origin, cultivation methods and manufacturing practices and so until more formal regulations are introduced for these food supplement products, buying organic may be the best option.

The many cases of substituted or adulterated herbal products purchased from a very high number of national marketplaces, where the labeled botanical ingredients did not match the chemically identified ones are, unfortunately, accompanied by other low-quality issues which additionally affect the safety and potential efficacy of commercial herbal products. As many as forty-one peer reviewed research articles, which have reported a case of adulteration among analyzed commercial samples, have also reported other quality issues which further lower the overall quality expected by their users and consumers. Additionally, another nineteen studies reported quality issues of the tested products without identifying any proof for their botanical identity adulteration. For the majority of herbal products reported, considerable variability of their labeled metabolic profile and/or content, such as the alkaloid content of “ma-huang” (*Ephedra sinica* Stapf) products (Gurley, 1998) or Menispermi Rhizoma (*Menispermum dauricum* DC) products (Liu et al., 2013b), selected triterpene glycosides and phenolic constituents in black cohosh (*A. racemosa*) products (Jiang et al., 2006) or the PAC content of cranberry products (Turbitt et al., 2020). Furthermore, aside of significant product-to-product variability, the marked differences of the content of individual flavonoids/flavonolignans in milk thistle (*Silybum marianum* (L.) Gaertn.) products have revealed quality difference also between different batches by the same manufacturers (Fenclova et al., 2019).

The peer-reviewed authentication results and the methods which were successfully employed to analyze commercial herbal products and significantly contribute to a better understanding of authenticity issues affecting the herbal industry and provides an as close-to-reality possible picture of the commercial herbal products’ authenticity as well as examples of techniques to be efficiently and accurately used for their authentication.

It is clear that chemical analysis alone can only identify existing problems. In order to prevent these problems from arising in the first place, better governance needs to be implemented along all stages of the supply chain. Regulation can help with this process but resources are scarce and real progress on quality is more achievable through having closer and more focused co-operation between the regulators and the producers, manufacturers and retailers of herbal products.
