# Supplementary material for: Chemical Authentication of Botanical Ingredients: A Review of Commercial Herbal Products
Source: Front Pharmacol. 2021 Apr 15;12:666850. doi: 10.3389/fphar.2021.666850 (PMC8082499; doi:10.3389/fphar.2021.666850)
Supplement: Supplementary file 1 [file table1.pdf]

Supplementary Material

Supplementary Table 1 | The authenticity of commercial herbal products at continental level

Continent	Countries	Products	Products/country	Authentic		Adulterated	
	no.	no.	$\bar{x}$	no.	%(*)	no.	%(*)
**Asia**	9	877	97.4	654	*75*	223	*25*
**N. America**	3	767	255.6	563	*73*	204	*27*
**Europe**	20	573	28.6	413	*72*	160	*28*
**S. America**	2	86	43	37	*43*	49	*57*
**Australia**	2	25	12.5	14	*56*	11	*44*
**Africa**	1	5	5	2	40	3	60

*The percentage values were rounded to the nearest whole number
